# The Missing Link between *Candida albicans* Hyphal Morphogenesis and Host Cell Damage

**DOI:** 10.1371/journal.ppat.1005867

**Published:** 2016-10-20

**Authors:** Duncan Wilson, Julian R. Naglik, Bernhard Hube

**Affiliations:** 1 Aberdeen Fungal Group, MRC Centre for Medical Mycology, School of Medicine, Medical Sciences and Nutrition, University of Aberdeen, Institute of Medical Sciences, Aberdeen, United Kingdom; 2 Mucosal & Salivary Biology Division, Dental Institute, King’s College London, United Kingdom; 3 Department of Microbial Pathogenicity Mechanisms, Hans Knoell Institute, and Friedrich Schiller University, Jena, Germany; Geisel School of Medicine at Dartmouth, UNITED STATES

## Introduction

Fungal pathogens are more commonly associated with morbidity and mortality than generally appreciated. In fact, a significant portion of the world population is infected by fungi, and an estimated 1.5 million people die from life-threatening fungal infections each year [1]. One of the most common fungal pathogens of humans is *Candida albicans*. The majority of the human population is colonised with this fungus, and superficial infections of mucosal surfaces are extremely common [2].

The morphological plasticity of *C*. *albicans* has long been implicated in the virulence of this pathogen [3]. The two most important morphologies, yeast and hyphal cells, are both required for virulence. Neither yeast-locked strains nor hyperfilamentous mutants are fully virulent in experimental systemic infections. However, it is generally accepted that each of the two forms fulfils specific functions during infection. While the yeast form is likely important for dissemination via the blood stream, the formation of filamentous hyphae contributes to adhesion and invasion of host cells.

## What’s Special about Hyphae?

**Fig 1:**
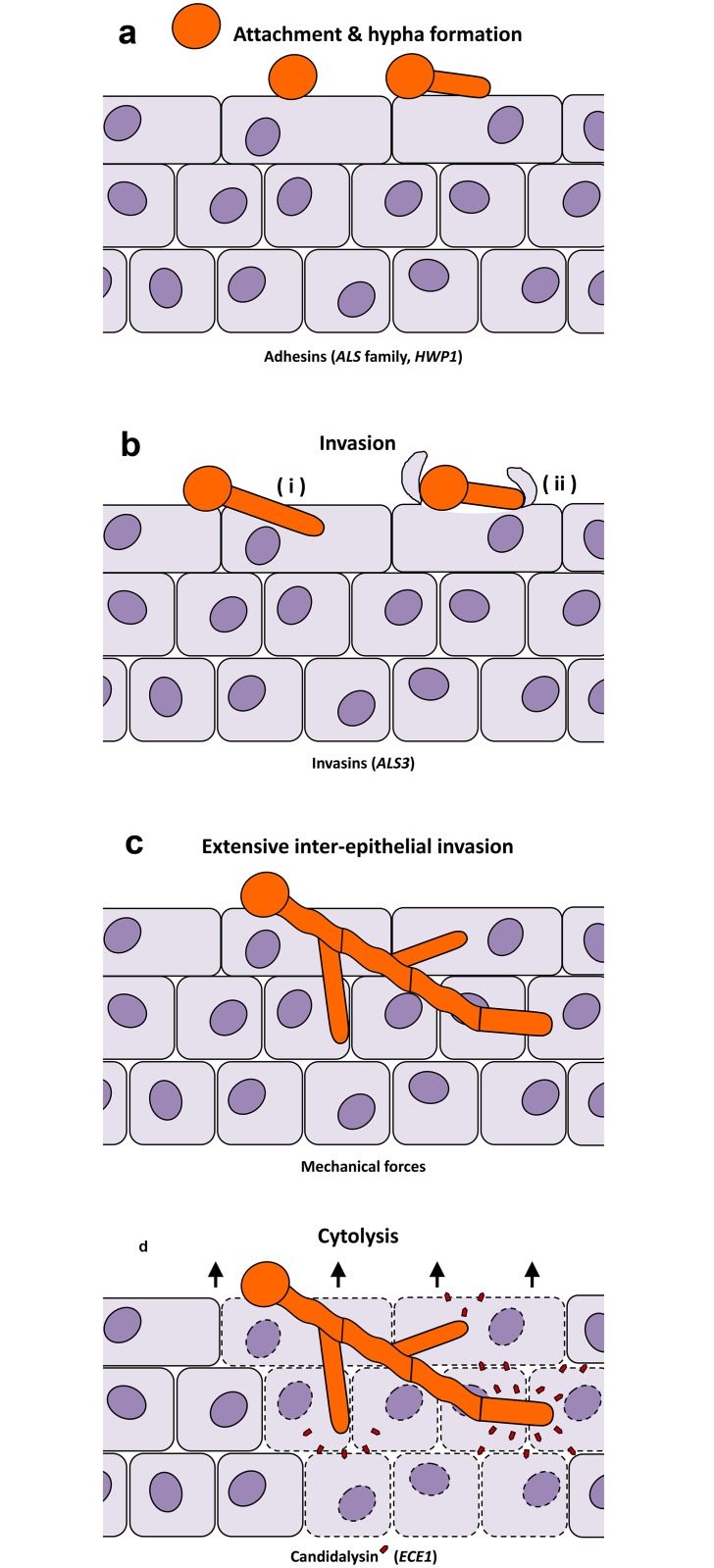
Distinct stages of *C*. *albicans*-epithelial infection. (**a**) In experimental epithelial infections, *C*. *albicans* yeasts form hyphae upon contact with epithelia and adhere tightly to the host cells. This is mediated by a number of adhesins, including members of the Als family and Hwp1. (**b**) This is followed by initial epithelial invasion via two routes—(i) fungal-driven active penetration and (ii) host-mediated induced endocytosis. (**c**) Elongating and branching hyphae result in extensive interepithelial invasion. Surprisingly, this invasion itself does not cause damage to the epithelium. (**d**) Simultaneous secretion of the fungal peptide toxin, Candidalysin (red pentagons), lyses the host epithelia and causes tissue destruction.

The invasive nature of hyphae is intuitive and supported by multiple studies (Fig 1). (i) Hyphae are the most common morphology observed during experimental infections and in patient biopsies, and histological analysis clearly shows that hyphae are the dominant invasive form [4]. (ii) Hyphae adhere more robustly and efficiently to host cells than yeast cells, largely owing to two hypha-associated adhesins, Als3 and Hwp1 [5] (Fig 1a). However, in certain environments, such as dynamic endothelial-interactions, yeast cells [6] or short germ tubes [7] have been reported to be more adherent than longer hyphae. (iii) Only hyphae invade efficiently into human cells, which occurs via two routes; induced endocytosis and active penetration [8] (Fig 1b). Induced endocytosis is mediated by the hypha-associated invasin, Als3, and is mainly dependent on host activities—even killed hyphae are endocytosed as long as Als3 is expressed on their surface. Active penetration, on the other hand, is a fungal-driven process that requires fungal viability but not host activity. Both invasion routes require hyphae, and mutants defective in hypha formation are also defective in host cell invasion [9]. However, hypha-mediated invasion of host cells by either route does not necessarily cause cell damage (Fig 1a–1c). Whilst *C*. *albicans* hyphae formation appears to play a central role in host tissue invasion, other morphotypes are critical during infections of other host niches. For example, yeast cell dispersal likely plays a key role in seeding the bloodstream from biofilms formed on indwelling medical devices [10]. (iv) Hyphal cells are involved in trace metal acquisition. During the transition from commensalism to invasion, *C*. *albicans* utilises different assimilation strategies to gain nutrients from host cells. Hyphae of *C*. *albicans* can efficiently bind the host iron storage protein ferritin [11] and host zinc [12] during invasion of epithelial or endothelial cells, promoting fungal growth. Notably, the *C*. *albicans* ferritin-binding protein is Als3, suggesting multiple virulence functions for this protein, including adhesion, invasion, and iron acquisition. The pH-regulated antigen 1 (Pra1) acts as a secreted zinc-binding protein and also possesses immune evasion functions via binding complement regulators and thereby avoiding complement deposition [13]. (v) Hyphae facilitate fungal escape from phagocytes and induce macrophage killing via a two-step mechanism: initiation of pyroptosis and piercing of the macrophage membrane [14]. (vi) Finally, the expression of other virulence-associated genes is linked to the morphological transition. These include hypha-associated secreted aspartyl protease genes (*SAP4-6*) [15] and the superoxide dismutase gene *SOD5* [16], but also a small set of eight core response genes, which are expressed under hypha-inducing conditions [17]. These hypha-associated virulence genes may have distinct functions for invasion processes and may prepare the invading fungal cells for impending host niches [18]. Therefore, hypha development is coupled to multiple invasion-associated properties, but if invasion per se does not directly damage host cells, how does this process occur?

## How Do Hyphae Damage Host Tissue?

As discussed above, hypha formation has long been known to be associated with a number of pathogenic properties and is a prerequisite for damage induction. However, the identification of a specific *C*. *albicans* factor that directly induces cell damage had remained elusive. This missing link between hyphal morphogenesis and damage induction has now been identified as a cytolytic toxin called Candidalysin, a 31 amino acid peptide [19] (Fig 1d). Candidalysin is generated from its parent protein, Ece1, which is encoded by the gene *ECE1*. *ECE1* is one of the eight core filamentation genes in *C*. *albicans* and was first discovered in the 1990s due to its high expression during hypha formation [20]. However, its molecular function remained unknown for almost a quarter of a century. In silico analysis suggested that Ece1 is a polypeptide consisting of a secretion signal peptide followed by eight short peptides, each separated by lysine/arginine residues. Previous studies had shown that these dibasic amino acids can be recognised by a subtilisin-like serine protease, Kex2, in the Golgi apparatus [21]. Proteomic analysis confirmed that Ece1 is produced by *C*. *albicans* hyphae and is sequentially processed at arginine/lysine residues by Kex2 and another serine protease, Kex1, respectively, followed by peptide secretion [19]. Candidalysin is one of these peptides. Candidalysin adopts an α-helical structure and, when secreted in sufficient quantities, intercalates and permeabilises host epithelial membranes to induce cell lysis. The presence of cholesterol in target membranes enhanced the lytic activity of Candidalysin, suggesting that membrane sterols may contribute to target specificity. Additional molecular analyses demonstrated the importance of Candidalysin, since deletion of only the Candidalysin-encoding region from the *ECE1* gene abolished the ability of *C*. *albicans* to damage epithelial cells in vitro and significantly attenuated *C*. *albicans* virulence in two in vivo models of mucosal infection: a cortisone acetate-treated mouse model of oropharyngeal candidiasis and a zebrafish swim bladder infection model [19]. Therefore, it appears that production of Candidalysin rather than hypha formation per se is the mediator of host cell damage. Given that Candidalysin is a hypha-associated factor, these observations finally provide the elusive missing link between filamentation and host cell damage and explain why *C*. *albicans* hyphae are the destructive morphology during mucosal infections. This work also identifies Candidalysin as one of the very few “classical virulence factors” in human pathogenic fungi [22].

## How Do Epithelial Cells Detect Candidalysin to Induce Immunity?

While Candidalysin is critical for fungal pathogenicity, our immune system is not helpless against this peptide toxin. Candidalysin is recognised by host epithelial cells and has been identified as the hyphal moiety that triggers the “danger response” pathway in epithelial cells. This pathway comprises NF-kB and PI3K signalling along with strong activation of MAPK signalling, resulting in activation of the transcription factor c-Fos via the p38 pathway and MKP1 via the ERK1/2 pathway [23,24]. Hence, when encountering yeast cells, our mucosal tissues tolerate these as benign colonisers but when encountering damage-inducing hyphae, Candidalysin induces the danger response pathway. In this way, the host is able to discriminate between the commensal and pathogenic states of *C*. *albicans*. These signalling events ultimately induce epithelial cytokine production and recruit immune cells (phagocytes and dendritic cells) to defend against infection. Intriguingly, the epithelial danger response has learned to respond to Candidalysin at levels below those required to induce cell lysis. For example, p-MKP1, c-Fos, and the nondamage-associated cytokine G-CSF were induced by sublytic concentrations (≤3 μM) of Candidalysin, and by a modified nontoxic version of the peptide. We propose that this dual function of Candidalysin is the result of a coevolutionary event; the fungus has developed an efficient peptide toxin to damage host membranes and, in response, the host has evolved a sensitive Candidalysin detection system to identify and defend itself against this common mucosal pathogen.

Given the worldwide prevalence of mucosal *C*. *albicans* infections [1], the identification of the first cytolytic peptide toxin produced by a human fungal pathogen has therapeutic potential for the treatment of mucosal candidiasis.

## References

[1] Brown GD, Denning DW, Gow NA, Levitz SM, Netea MG, White TC (2012) Hidden killers: human fungal infections. Sci Transl Med 4: 165rv113 10.1126/scitranslmed.3004404 23253612

[2] Fidel PL Jr. (2007) History and update on host defense against vaginal candidiasis. Am J Reprod Immunol 57: 2–12. 10.1111/j.1600-0897.2006.00450.x 17156186

[3] Sudbery PE (2011) Growth of *Candida albicans* hyphae. Nat Rev Microbiol 9: 737–748. 10.1038/nrmicro2636 21844880

[4] Odds F (1988) *Candida* and candidosis. 2nd edition, Bailliere Tindall, London, UK.

[5] Mayer FL, Wilson D, Hube B (2013) *Candida albicans* pathogenicity mechanisms. Virulence 4. 10.4161/viru.22913 PMC3654610 23302789

[6] Grubb SE, Murdoch C, Sudbery PE, Saville SP, Lopez-Ribot JL, Thornhill MH (2009) Adhesion of *Candida albicans* to endothelial cells under physiological conditions of flow. Infect Immun 77: 3872–3878. 10.1128/IAI.00518-09 19581400 PMC2738003

[7] Wilson D, Hube B (2010) Hgc1 mediates dynamic *Candida albicans*-endothelium adhesion events during circulation. Eukaryot Cell 9: 278–287. 10.1128/EC.00307-09 20023069 PMC2823009

[8] Wachtler B, Citiulo F, Jablonowski N, Forster S, Dalle F, Schaller M, Wilson D, Hube B (2012) *Candida albicans*-epithelial interactions: dissecting the roles of active penetration, induced endocytosis and host factors on the infection process. PLoS ONE 7: e36952 10.1371/journal.pone.0036952 22606314 PMC3351431

[9] Wachtler B, Wilson D, Haedicke K, Dalle F, Hube B (2011) From attachment to damage: defined genes of *Candida albicans* mediate adhesion, invasion and damage during interaction with oral epithelial cells. PLoS ONE 6: e17046 10.1371/journal.pone.0017046 21407800 PMC3044159

[10] Uppuluri P, Chaturvedi AK, Srinivasan A, Banerjee M, Ramasubramaniam AK, Kohler JR, Kadosh D, Lopez-Ribot JL (2010) Dispersion as an important step in the *Candida albicans* biofilm developmental cycle. PLoS Pathog 6: e1000828 10.1371/journal.ppat.1000828 20360962 PMC2847914

[11] Almeida RS, Brunke S, Albrecht A, Thewes S, Laue M, Edwards JE, Filler SG, Hube B (2008) The hyphal-associated adhesin and invasin Als3 of *Candida albicans* mediates iron acquisition from host ferritin. PLoS Pathog 4: e1000217 10.1371/journal.ppat.1000217 19023418 PMC2581891

[12] Citiulo F, Jacobsen ID, Miramon P, Schild L, Brunke S, Zipfel P, Brock M, Hube B, Wilson D (2012) *Candida albicans* scavenges host zinc via Pra1 during endothelial invasion. PLoS Pathog 8: e1002777 10.1371/journal.ppat.1002777 22761575 PMC3386192

[13] Zipfel PF, Skerka C, Kupka D, Luo S (2011) Immune escape of the human facultative pathogenic yeast *Candida albicans*: the many faces of the *Candida* Pra1 protein. Int J Med Microbiol 301: 423–430. 10.1016/j.ijmm.2011.04.010 21565550

[14] Krysan DJ, Sutterwala FS, Wellington M (2014) Catching fire: *Candida albicans*, macrophages, and pyroptosis. PLoS Pathog 10: e1004139 10.1371/journal.ppat.1004139 24967821 PMC4072798

[15] Naglik JR, Challacombe SJ, Hube B (2003) *Candida albicans* secreted aspartyl proteinases in virulence and pathogenesis. Microbiol Mol Biol Rev 67: 400–428, table of contents. 10.1128/MMBR.67.3.400-428.2003 12966142 PMC193873

[16] Martchenko M, Alarco AM, Harcus D, Whiteway M (2004) Superoxide dismutases in *Candida albicans*: transcriptional regulation and functional characterization of the hyphal-induced *SOD5* gene. Mol Biol Cell 15: 456–467. 10.1091/mbc.E03-03-0179 14617819 PMC329211

[17] Martin R, Albrecht-Eckardt D, Brunke S, Hube B, Hunniger K, Kurzai O (2013) A Core Filamentation Response Network in *Candida albicans* Is Restricted to Eight Genes. PLoS ONE 8: e58613 10.1371/journal.pone.0058613 23516516 PMC3597736

[18] Brunke S, Hube B (2014) Adaptive prediction as a strategy in microbial infections. PLoS Pathog 10: e1004356 10.1371/journal.ppat.1004356 25275642 PMC4183746

[19] Moyes DL, Wilson D, Richardson JP, Mogavero S, Tang SX, Wernecke J, Hofs S, Gratacap RL, Robbins J, Runglall M, Murciano C, Blagojevic M, Thavaraj S, Forster TM, Hebecker B, Kasper L, Vizcay G, Iancu SI, Kichik N, Hader A, Kurzai O, Luo T, Kruger T, Kniemeyer O, Cota E, Bader O, Wheeler RT, Gutsmann T, Hube B, Naglik JR (2016) Candidalysin is a fungal peptide toxin critical for mucosal infection. Nature. 532: 64–8 10.1038/nature17625 27027296 PMC4851236

[20] Birse CE, Irwin MY, Fonzi WA, Sypherd PS (1993) Cloning and characterization of *ECE1*, a gene expressed in association with cell elongation of the dimorphic pathogen *Candida albicans* . Infect Immun 61: 3648–3655. 8359888 10.1128/iai.61.9.3648-3655.1993 PMC281060

[21] Bader O, Krauke Y, Hube B (2008) Processing of predicted substrates of fungal Kex2 proteinases from *Candida albicans*, *C*. *glabrata*, *Saccharomyces cerevisiae* and *Pichia pastoris* . BMC Microbiol 8: 116 10.1186/1471-2180-8-116 18625069 PMC2515848

[22] Casadevall A, Pirofski LA (2014) Microbiology: Ditch the term pathogen. Nature 516: 165–166. 10.1038/516165a 25503219

[23] Moyes DL, Runglall M, Murciano C, Shen C, Nayar D, Thavaraj S, Kohli A, Islam A, Mora-Montes H, Challacombe SJ, Naglik JR (2010) A biphasic innate immune MAPK response discriminates between the yeast and hyphal forms of *Candida albicans* in epithelial cells. Cell Host Microbe 8: 225–235. 10.1016/j.chom.2010.08.002 20833374 PMC2991069

[24] Moyes DL, Shen C, Murciano C, Runglall M, Richardson JP, Arno M, Aldecoa-Otalora E, Naglik JR (2014) Protection against epithelial damage during *Candida albicans* infection is mediated by PI3K/Akt and mammalian target of rapamycin signaling. J Infect Dis 209: 1816–1826. 10.1093/infdis/jit824 24357630 PMC4017362

